# Nanostructure of Clustered DNA Damage in Leukocytes after In-Solution Irradiation with the Alpha Emitter Ra-223

**DOI:** 10.3390/cancers11121877

**Published:** 2019-11-26

**Authors:** Harry Scherthan, Jin-Ho Lee, Emanuel Maus, Sarah Schumann, Razan Muhtadi, Robert Chojowski, Matthias Port, Michael Lassmann, Felix Bestvater, Michael Hausmann

**Affiliations:** 1 Bundeswehr Institute of Radiobiology, University of Ulm, Neuherbergstraße 11, 80937 München, Germany; jin-ho.lee@kip.uni-heidelberg.de (J.-H.L.); Emanuel_Maus@gmx.net (E.M.); Razan1Muhtadi@bundeswehr.org (R.M.); MatthiasPort@bundeswehr.org (M.P.); 2 Kirchhoff-Institute for Physics, Heidelberg University, Im Neuenheimer Feld 227, 69120 Heidelberg, Germany; chojowski@stud.uni-heidelberg.de; 3 Department of Nuclear Medicine, University of Würzburg, Oberdürrbacher Str. 6, 97080 Würzburg, Germany; Schumann_S1@ukw.de (S.S.); Lassmann_M@ukw.de (M.L.); 4 German Cancer Research Center (DKFZ), Im Neuenheimer Feld 280, 69120 Heidelberg, Germany; f.bestvater@dkfz.de

**Keywords:** complex DNA damage, DNA repair, high LET irradiation, Single Molecule Localization Microscopy (SMLM), DSB focus substructure

## Abstract

Background: Cancer patients are increasingly treated with alpha-particle-emitting radiopharmaceuticals. At the subcellular level, alpha particles induce densely spaced ionizations and molecular damage. Induction of DNA lesions, especially clustered DNA double-strand breaks (DSBs), threatens a cell’s survival. Currently, it is under debate to what extent the spatial topology of the damaged chromatin regions and the repair protein arrangements are contributing. Methods: Super-resolution light microscopy (SMLM) in combination with cluster analysis of single molecule signal-point density regions of DSB repair markers was applied to investigate the nano-structure of DNA damage foci tracks of Ra-223 in-solution irradiated leukocytes. Results: Alpha-damaged chromatin tracks were efficiently outlined by γ-H2AX that formed large (super) foci composed of numerous 60–80 nm-sized nano-foci. Alpha damage tracks contained 60–70% of all γ-H2AX point signals in a nucleus, while less than 30% of 53BP1, MRE11 or p-ATM signals were located inside γ-H2AX damage tracks. MRE11 and p-ATM protein fluorescent tags formed focal nano-clusters of about 20 nm peak size. There were, on average, 12 (±9) MRE11 nanoclusters in a typical γ-H2AX-marked alpha track, suggesting a minimal number of MRE11-processed DSBs per track. Our SMLM data suggest regularly arranged nano-structures during DNA repair in the damaged chromatin domain.

## 1. Introduction

Ionizing radiation (IR) transfers energy to electrons of atoms and molecules, leading to their ionization. Particle irradiation by, e.g., alpha-emitting radionuclides displays high linear energy transfer (LET) to the molecular and supra-molecular environment along a particle track through the cell and its nucleus. High LET radiation leads to densely accumulated ionization events and consequently molecular damage along several µm and within some ten nm perpendicular to the track [1,2,3]. 

Systemic patient treatment with alpha particle-emitting radiopharmaceuticals is increasingly being applied in standard care for patients with metastatic castrate-resistant prostate cancer [4,5]. Alpha particles considered for treatment have a range of up to 90 µm in water [6] and they have a kinetic energy of about 1.4 to 2.2 MeV per nucleon. They pass cells with a mean LET of about 100 keV µm^−1^ [6]. While the alpha particles lose energy and slow down when traversing matter, their interaction probability increases. The LET of alpha particles peaks towards the end of their tracks at the so-called Bragg peak with a LET of about 220 keV µm^−1^ [6]. Ra-223 is an α-emitter with a half-life of 11.43 days and is known to decay in six steps [see e.g. Ref. [7]] into stable Pb-207. The progeny Rn-219, Po-215 and Bi-211 are α-emitters as well, so that in total four α-particles will be emitted per Ra-223 decay, depositing energies between 5.77 MeV (Ra-223) and 7.49 MeV (Po-215). Since the decay yield of Bi-211 (6.66 MeV) into Po-211 amounts to less than 0.3% it is disregarded. Considering this branch, an additional α-particle is emitted when Po-211 decays into Pb-207 [7,8]. Dense ionizations and secondary electrons induce clustered damage along Ra-223-induced alpha trajectories in cells and their nuclei, including oxidative base damage, DNA crosslinks, and densely spaced DNA single and double strand breaks (DSBs) [9]. Such complex DNA damages confront the DNA repair machineries with hardly solvable tasks [10,11,12]. Therefore, complex DNA damage seeded by high LET irradiation efficiently induces cell death and mutagenesis as indicated by a high radiobiological effectiveness (RBE) [13]. 

In target cell nuclei, alpha irradiation induces clustered DNA lesions at the nm scale that are preferentially repaired by the non-homologous end joining (NHEJ) pathway(s) (canonical or alternative NHEJ) that predominates in euchromatin and G1/early S phase, while c-NHEJ and homologous recombination (HR) are at work in heterochromatin [14,15] and G2 phase (for review see [16]). The DNA damage response after particle irradiation involves DNA damage-responsive kinase signaling (ATM, ATR, DNA-PKcs) with the kinases being activated after Ku (DNA-PKcs) or NBS1 (ATM) binding to the DNA ends [17,18]. Additionally, alternative NHEJ that employs PARP1 and the MRN complex [16] is expected to play a role in processing of complex DNA breaks in the absence of c-NHEJ components, but it may only play a minor role in the cells studies, since c-NHEJ is the predominant NHEJ pathway in human G1 cells [19,20]. Active ATM, on the other hand, phosphorylates, among other downstream targets, the histone 2 variant H2AX at its Serin 139 moiety, commonly known as γ-H2AX [21]. γ-H2AX histone clusters can be visualized as microscopically visible immunofluorescent foci extending about 1MB around a DSB [22,23]. Various proteins assemble at the so-marked damaged chromatin domain and promote, or take part in, DSB repair [24]. Among these are 53BP1 (pathway decision), MRE11 (resection), RAD51 (single strand binding protein in the HR repair pathway) and other downstream targets [18,25,26,27]. 

After high LET particle irradiation and immunofluorescent demarcation of the DNA damage tracks, the structural nature and the distribution of DSB sites and associated DNA repair complexes along high-LET-induced damage tracks over a large range of LETs (LET > 100–1800 keV µm^−1^) remains largely obscure [28,29]. However, substructures (clusters) of DNA damage-associated and repair proteins (which often serve as DSB surrogate markers) have been revealed for high and low LET irradiation by super-resolution light microscopy [30,31,32,33] and transmission electron microscopy (TEM) [3,34]. DNA damage foci are large MB chromatin regions that harbor phosphorylated H2AX molecules that resolve as sub- or nano-foci of about 100 nm consisting of even smaller signal accumulations [30,32] after 10 Gy photon irradiation, with the spatial organization of nano-foci being dependent on the chromatin insulator CTCF [33]. Based on TUNEL 3′-DNA end labeling and Ku70 staining, the latter authors reported that large asymmetric γ-H2AX cluster-like structures of about 500 nm (hereafter called super-foci) in low LET-irradiated chromatin contain single DSBs [33]. At the TEM level, micro-clusters of DNA repair proteins have been detected along heavy ion-induced damage tracks in nuclear chromatin, suggesting the possibility that up to 500 DSBs/µm^3^ may be concentrated in a carbon ion track [34]. However, whether the distribution of the repair micro-clusters studied directly reflects DSBs remains to be further investigated. 

Due to the growing interest in systemic radiotherapies with alpha emitters in clinical practice, we recently characterized the DNA damage incurred after low dose (< 150 mGy) internal ex-vivo exposure of peripheral blood and its lymphocytes to the alpha emitters Ra-223 and Ra-224 [8,35]. Ra-223 dichloride, also known as Alpharadin (Xofigo^®^), is applied in the therapy of patients with metastatic castration-resistant prostate cancer. In these ex-vivo in-solution irradiation studies, a calibration for DNA damage elicited by dissolved Ra-223 chloride and Ra-224-dichloride was performed, revealing that the number of cells traversed by an alpha particle correlates linearly with the absorbed dose to the blood, with about every 5th cell carrying a single alpha particle track after ex-vivo exposure of 100 mGy absorbed dose to the blood [8,35]. 

In this contribution, we used Single Molecule Localization Microscopy (SMLM) to investigate the spatial organization of damaged chromatin regions and the recruitment of repair-related proteins along alpha tracks in Ra-223-exposed leukocyte nuclei at ultra-resolution. SMLM can detect single fluorescent molecules and localizes them with nm precision. Images reflecting the coordinates of the molecules result in an optical resolution (= smallest resolvable point-to-point distance) down to the order of about 10 nm in IR-exposed cell nuclei [36,37]. SMLM is based on the concept of using fluorescent labels that can be switched between two different spectral excitation states (e.g., off/on) to achieve temporal isolation and thus spatial separation of molecular signals [38]. The fluorescent molecules randomly return from an induced dark state back to the emission state, which takes place over a time regime orders of magnitude slower than the fluorescent emission decay. By this stochastic blinking, the determination of the center-of-mass (barycenter) of an Airy disc of a single molecule image approximates the location of the emitting molecule, so that a coordinate matrix of all fluorescent molecules is obtained, making it possible to calculate distances and structural parameters based on Ripley’s point-to-point metrics [39]. After data analysis, artificial “pointillist” super-resolution images can be generated, in which the results of data analysis can additionally be encoded [40]. SMLM was applied to investigate the irradiation effects on chromatin nano-architecture [28,36,37] and protein organization at the nano scale [41,42,43], revealing systematic dose- [40,44] and locus-dependent [45] chromatin conformation changes. For multi-color localization microscopy of DNA damage markers along alpha tracks, we here applied molecular labelling with specific antibodies against γ-H2AX, MRE11, and 53BP1 in alpha-traversed cell nuclei and studied their distribution within γ-H2AX foci domains (super-foci) that demarcate the particle track.

## 2. Results

### 2.1. Distribution of DNA Damage-Associated Proteins in Ra-223-Irradiated Leukocyte Nuclei

Peripheral blood, as an easily accessible tissue, is frequently used in biodosimetric techniques. Here, we investigated the molecular damage in the nuclei of ethanol-fixed leukocytes traversed by α-particles [8] after immunostaining for the DNA damage-associated Serin-139-phosphorylated histone H2AX, (γ-H2AX) [21]. γ-H2AX marks about one Mb chromatin domain around a DSB where DNA damage sensor and repair proteins accumulate as microscopically visible foci [22,24,46,47]. 

**Figure 1:**
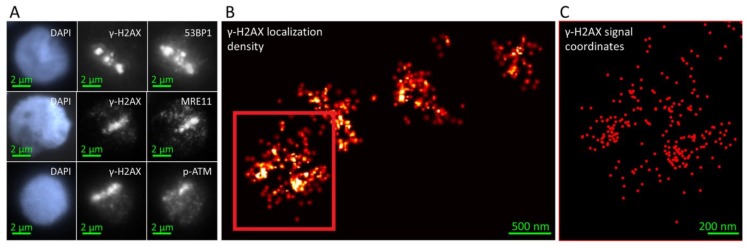
Fluorescence microscopy of γ-H2AX and DNA damage response proteins 53BP1, MRE11 and p-ATM in a single alpha-trajectory. (**A**) Wide-field overview images of human leukocyte nuclei stained with DAPI (blue), γ-H2AX and different proteins (53BP1, MRE11, p-ATM) showing typical regions of interests used for SMLM image acquisition. (**B**) Representative SMLM signal point density image of a leukocyte nucleus showing several large γ-H2AX super-foci linearly aligned along the trajectory of a single alpha particle. Slice thickness 500 nm. (**C**) Magnification and signal coordinate representation of red box in (**B**) showing that one γ-H2AX damage super-focus consists of substructures and smaller regions of high signal densities at the nanoscale.

Alpha particle-induced DNA damage tracks appear at the wide-field light microscopy level as linear streaks of successions of globular γ-H2AX-positive chromatin domains or large foci (super-foci) (Figure 1A). γ-H2AX formation is largely dependent on the DNA double strand break (DSB)-responsive kinase ATM that in its active form is phosphorylated at Serin-1981 [48], which depends on complex formation with NBS1 [49]. Wide-field fluorescence microscopy revealed that the damaged chromatin regions along the alpha particle tracks contained large amounts of the DNA damage sensor protein 53BP1 (Figure 1A), while the DNA repair processive nuclease MRE11 [50] was present at lower frequency. The activated phos-S1981(p)ATM kinase was only seen as minute foci throughout the γ-H2AX-positive chromatin tracks.

**Figure 2:**
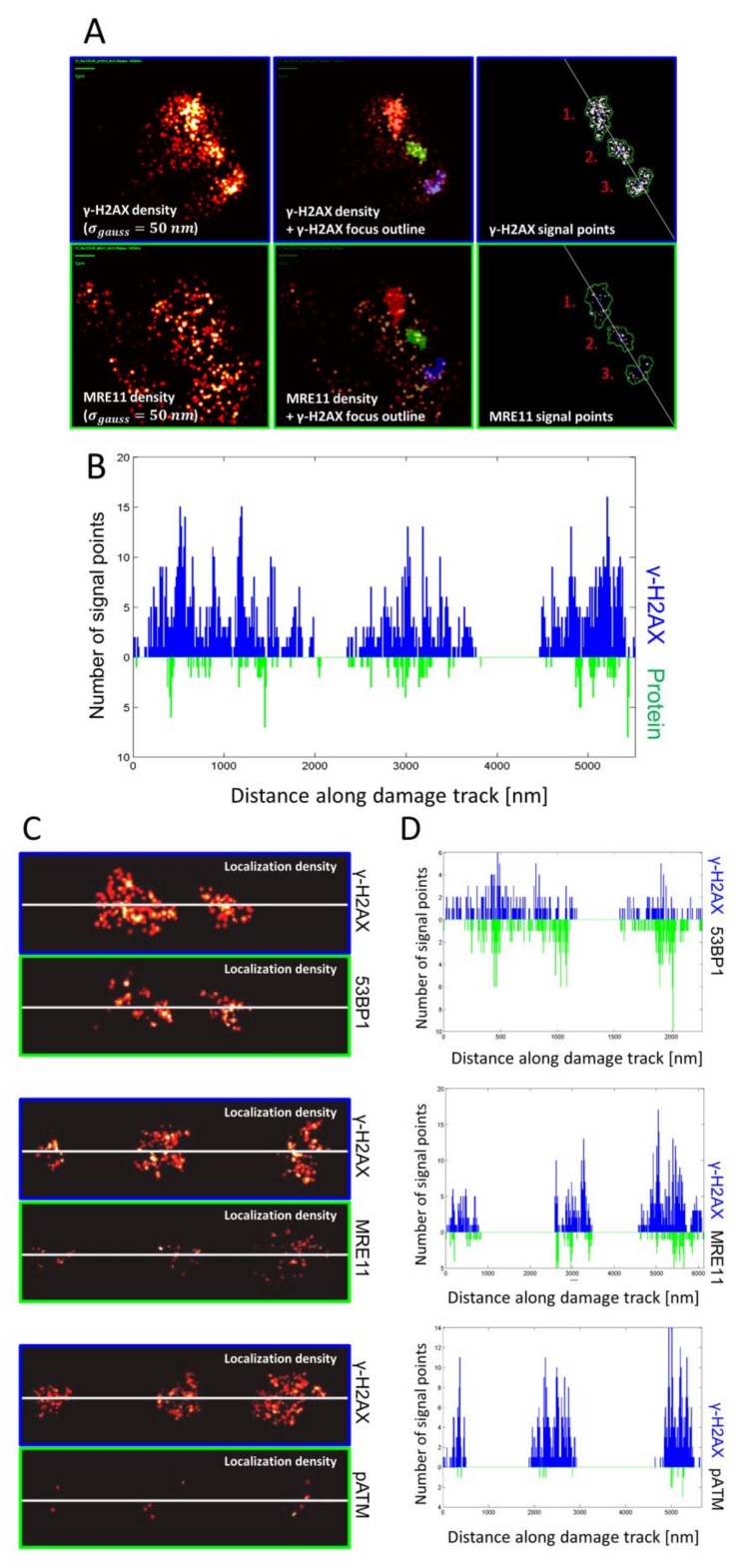
Masking and track nanostructure obtained by γ-H2AX and DNA damage response protein (53BP1, MRE11 and p-ATM) signal tagging. (**A**) Visualization of representative γ-H2AX (top left) and DSB-associated protein (MRE11; bottom left) signal point densities (blurred with gauss filter with σ_gauss_ = 50 nm. DBSCAN analysis of γ-H2AX signal points using ε = 200 nm and adjusted minimum point number of 25. Overlay of γ-H2AX (top middle) and protein signal densities (bottom middle) with corresponding γ-H2AX foci outline (red/green/blue) show the relative microscale distribution along the damage track. γ-H2AX (top-right) and protein (bottom-right) localization signals were masked with corresponding γ-H2AX foci areas. Where necessary, the alpha particle trajectory was approximated by a linear fit (white line) through the barycenter coordinates of each focus. The underlying signal point distribution of γ-H2AX and respective protein was calculated perpendicularly along the fit curve. (**B**) Histogram of signal number distribution of γ-H2AX (blue) and an example protein (green) inside γ-H2AX super-foci along an estimated alpha particle trajectory derived as shown in (**A**), with the first signal point along the linear trajectory being set to the distance value of 0. In this example, the start-to-end orientation of damage tracks was arranged arbitrarily. (**C**) Illustrations of localization density images of γ-H2AX damage tracks (masked with the corresponding γ-H2AX super-foci outlines) co-stained with 53BP1 (top), MRE11 (middle) and p-ATM (bottom) along the estimated alpha trajectory lines (white lines). (**D**) Signal number histogram plots along the masked particle tracks showing the signal numbers for each γ-H2AX/protein pair depicted in (**C**). Overall, 53BP1 signal numbers were comparable or in some cases even higher than γ-H2AX signal numbers (top), with the distribution of signal points along the particle track showing strong correlation between γ-H2AX and 53BP1. Signal numbers for MRE11 were significantly lower than for γ-H2AX. Again, small peak regions of high signal densities seemed to occur between MRE11 and γ-H2AX, thereby revealing the presence of smaller complex sub-structures. p-ATM signal points are sparsely distributed along the damage track, but co-occurred with regions dense in γ-H2AX signals.

To reveal the DNA damage- and repair-associated protein distribution within the damage-loaded chromatin tracks, we applied SMLM (Figure 1B,C) and quantitative analyses of chromatin conformations including statistical physics, graph theory and cluster algorithms to study repair protein arrangements. As noted by wide-field microscopy (Figure 1A), alpha-induced DNA damage tracks in leukocyte nuclei showed several, more-or-less linearly aligned super-foci or continuous tracks that consisted of highly concentrated γ-H2AX fluorescent tags that represented the most abundant damage marker (Figure 1B and Figure 2A), as expected from earlier analysis [8,35,46,51,52]. At an even higher resolution, the super-foci could be subdivided into further subunits with characteristic signal accumulations (Figure 1 and Figure 2). On the nano-scale, using SMLM, the molecular signal point pattern obtained by resolving each large γ-H2AX super-focus along an alpha particle damage track consisted of several smaller sub-regions of numerous closely spaced γ-H2AX single molecule signals that represent nano-foci or -clusters [30,33,36]. For quantification of these nano-clusters and the distribution patterns of the single molecule signals of the other DSB repair-related proteins (53BP1, MRE11, p-ATM) we computed density-based γ-H2AX super-focus-outlines from the SMLM data and used these to mask the signal point coordinate matrix of the nuclei (Figure 2A, upper row) by transfer as reference areas (Figure 2A, lower row) to study the in-track distribution of the other DNA-damage-associated proteins (Figure 2B). Analysis parameters were optimized by defining specific cut-off of points inside a given radius so that the contours of foci include regions of high γ-H2AX signal point densities matching the outlines of the γ-H2AX-marked super-foci or damage tracks at the micro scale (see Materials & Methods; Figure 2A,B). In nuclei with 2–4 super-foci, the original α-particle track could be approximately reconstructed by a linear fit through the foci barycenters (Figure 2A,C). The distribution of single molecule signals perpendicular to the track was then displayed quantitatively along the direction of the particle trajectory for γ-H2AX, 53BP1, MRE11 and p-ATM (Figure 2D). 

The frequency plots along the particle tracks locally show the signal numbers of γ-H2AX and the numbers of the analyzed proteins for each cell nucleus analyzed. 53BP1 signal numbers were similar to the γ-H2AX signal numbers. Signal numbers for MRE11 were always lower than for γ-H2AX. p-ATM signal points co-occurring with γ-H2AX dense regions are sparsely distributed. 

### 2.2. Nano-Distribution of γ-H2AX, 53BP1, MRE11 and p-ATM in Alpha Tracks

**Figure 3:**
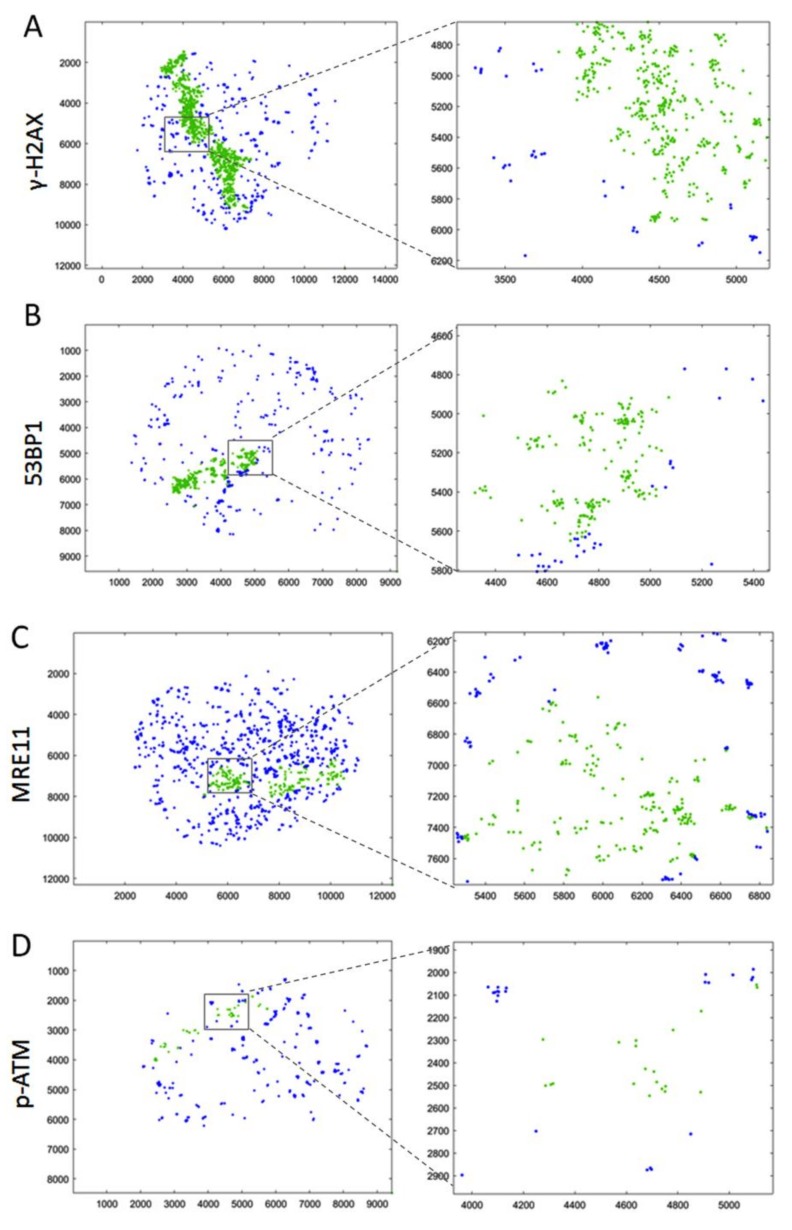
SMLM data point coordinates of γ-H2AX and 53BP1, MRE11 and p-ATM in alpha-trajectories. Representation of SMLM signal point positions in Cartesian coordinates over the whole nucleus for (**A**) γ-H2AX, (**B**) 53BP1, (**C**) MRE11, and (**D**) p-ATM. Following the masking procedure applied (c.f. Figure 2A), SMLM signal point coordinates are differentially colored for those present in γ-H2AX-marked damage track mask (green) and those detected throughout the nucleus (blue). The small grey-boxed sub-regions are blown up for better display to the right.

**Figure 4:**
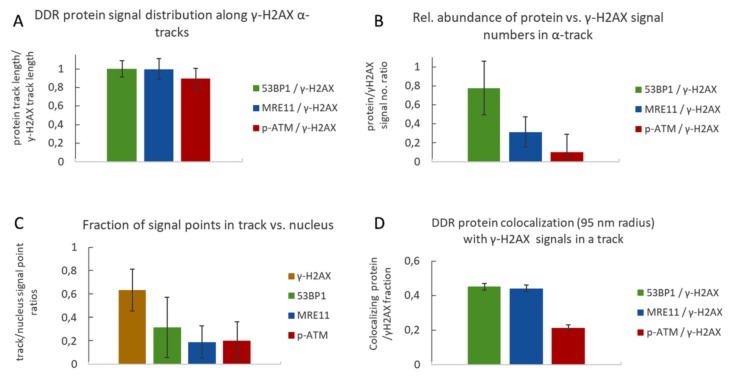
Quantitative analysis of γ-H2AX and 53BP1, MRE11 and p-ATM signals in damage tracks. (**A**) Relative frequency distribution of the DNA damage response (DDR) proteins along alpha tracks. The extension of a track was defined as the distance of the farthest signal points along the axis in a damage track signal number histogram (e.g., Figure 2). It appears that the average track length computed for γ-H2AX, 53BP1, MRE11 and p-ATM signals is similar, indicating that protein signals are distributed over the full extension of the damaged chromatin along the alpha particle trajectory. (**B**) Average ratios of DDR protein signal numbers relative to γ-H2AX signal numbers per average alpha track. In all experiments, γ-H2AX signal points were most frequent, followed in decreasing order by 53BP1 > MRE11 > p-ATM. (**C**) Ratio of signal point number abundance for γ-H2AX, 53BP1, MRE11 and p-ATM inside the average γ-H2AX alpha-track relative to signals over the nucleus. The signal numbers for γ-H2AX in different co-staining experiments were similar; thus, all γ-H2AX data were pooled for further single-color analyses. The average ratios of signal points detected inside the respective γ-H2AX damage track mask versus signal points detected over the whole nucleus show that most γ-H2AX signals are concentrated inside the damage track, which is less so for 53BP1, MRE11 and p-ATM. (**D**) Fraction of DDR protein signal points co-localizing with γ-H2AX signal points within a defined radius of 95 nm. 45% of 53BP1 and MRE11 signals in a track co-localize with γ-H2AX signals in a 95 nm radius, while only 21% of p-ATM signals showed co-localization. Error bars represent standard deviation.

SMLM data acquisition results in a matrix of coordinates that map the positions of the individually labelled single-molecule signals. Figure 3 depicts typical results, with the signals points contained in the γ-H2AX-masked alpha track displayed in green, while blue marks the signals in the surrounding chromatin of the nucleus. The accumulation of tags was then further resolved into single points with a localization resolution given by the precision of the SMLM measurement (about 15 nm in the experiments described here). SMLM data analysis of all damage tracks (*n* = 137) investigated for the distribution of a given protein along γ-H2AX tracks revealed the presence of 53BP1, MRE11 and p-ATM signals along the entire length of a given trajectory (Figure 4A), although the absolute number of signals was significantly different. On average, the γ-H2AX super-foci along a damage track contained 60–70% of all γ-H2AX point signals present in an alpha-traversed nucleus (Figure 4B). In contrast, only 20–30% of all 53BP1, MRE11 or p-ATM signals in a nucleus were located inside the masked damage tracks (Figure 4C). The presence of most nuclear γ-H2AX signal points along the damage track agrees with de novo formation of the S-139 phosphorylation-mark at H2AX histone molecules upon IR-induced dsDNA damage, while the applied MRE11 and 53BP1 antibodies used will also detect proteins outside the damaged chromatin domain.

Relative to γ-H2AX, the 53BP1 protein was most abundant (78% of γ-H2AX), followed by MRE11 (31%), while p-ATM displayed the lowest frequency (10%) in the γ-H2AX-marked super-foci outlines along a track (Figure 4B). These observations are consistent with 53BP1 being abundant throughout particle-induced chromatin tracks [3,31] and our wide-field observations (not shown). Still, a part of 53BP1 and MRE11 protein signals remained scattered throughout a nucleus with a single alpha particle hit. Activated p-S1981-ATM signals were also present along the damage tracks (Figure 4A) and showed the lowest abundance in the track compared to all other signals (Figure 4B), possibly reflecting the transient nature of active ATM/ damaged chromatin interactions. The level of pATM inside the track was with about 20% comparable to the level of MRE11 (Figure 4C). The low frequency of p-ATM within γ-H2AX-defined alpha tracks may be related to a limited temporal presence of the active kinase in the alpha-damaged chromatin tracks and/or partial loss of this protein during the preparation procedures [27]. In alpha-hit cells, there were also numerous p-ATM signals ubiquitously distributed throughout the cell nucleus (Figure 3D). This may be related to the dispersion of active p-ATM throughout the chromatin, where p-ATM and DNA-PKcs can transiently induce pan-nuclear γ-H2AX formation upon particle irradiation [53,54]. 

**Figure 5:**
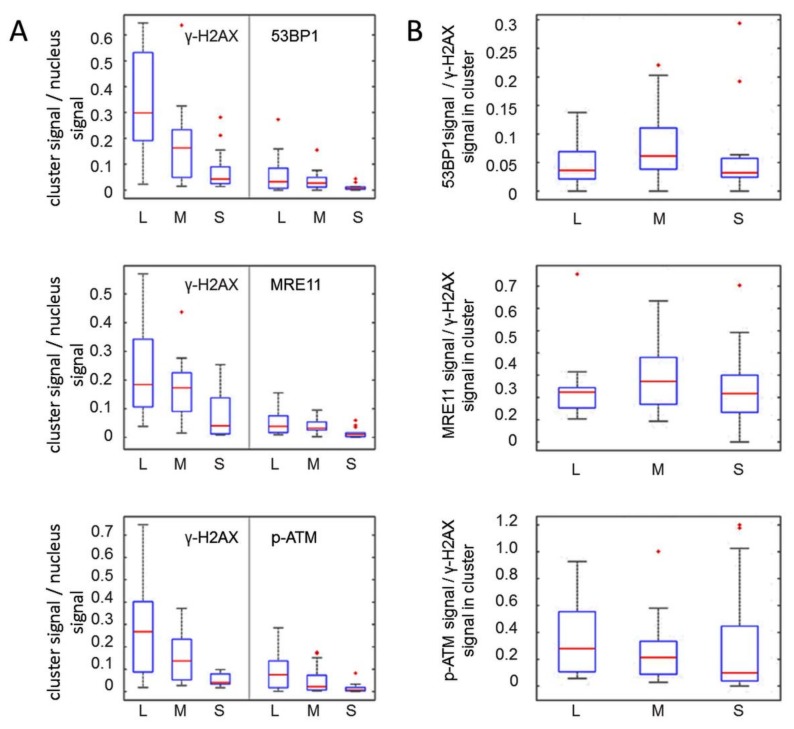
Orientation of γ-H2AX damage tracks consisting of at least three differently sized super-foci. (**A**) Analysis of γ-H2AX damage tracks consisting of at least three separate super-foci of decreasing size (*n* = 53) oriented from largest (L), to medium (M), to smallest (S). The signal points inside each focus were normalized against the total measured signal numbers. In cases with more than one focus in between the largest and the smallest focus along the damage track (*n* = 11), the average signal points per cluster was calculated. The results are presented as boxplots for γ-H2AX together with the respective co-stained 53BP1, MRE11 and p-ATM protein tags. (**B**) Boxplot of the ratios of 53BP1, MRE11 and p-ATM signal numbers (top to bottom, respectively) against corresponding co-stained γ-H2AX signals in L, M and S foci as defined in (A). The relative γ-H2AX/protein signal ratios remain relatively constant for all cluster sizes.

When we linearly aligned alpha-induced foci tracks marking the particle trajectory, we in some cases noted large, gradually sized γ-H2AX super-foci. The latter may be related to different chromatin compartments traversed by the particle, i.e., eu- and heterochromatin, which in turn may influence the propagation of the γ-H2AX chromatin signal [27], with progression of DNA repair in heterochromatin being slower and requiring its unfolding [14,15,27,36,55]. Different sizes of super-foci, on the other hand, may also be related to a different degree of damage. Selecting cell nuclei with gradually sized γ-H2AX super-foci retrieved 53 cells with a larger focus at one track end, indicating increased damage load, likely created by large energy deposition possibly near the Bragg peak (Figure 5A), and/or reflecting the local chromatin environment. These cell nuclei were separately analyzed in order to see whether this presumptive Bragg peak damage may be related to an altered DDR protein distribution compared to the rest of the track. 

It appeared that the largest super-focus in a nucleus also contained the largest number of the respective damage responsive protein signal points (Figure 5A), while the ratio of γ-H2AX and respective DNA damage-associated protein signal numbers remained nearly constant in the differently sized super-foci of a track (Figure 5B). Since most foci appear within 0.5 to 1 h post irradiation (e.g., [1,22]) and our irradiation scheme involved 1 h of in solution exposure, the data above indicate that the size variations of γ-H2AX super-foci directly relate to damage load.

### 2.3. Nano-Architecture of 53BP1, MRE11 and p-ATM in γ-H2AX-Outlined Damage Tracks

To investigate the molecular architecture of the γ-H2AX-marked chromatin regions further, we determined the co-occurrence (juxta-positioning) of the individual single molecule signal points by co-localization analysis (see Materials and Methods). The average “co-localization” radius of 95 nm between γ-H2AX and its co-stained proteins was in good agreement with previous super-resolution studies that detected a 100 nm radius of γ-H2AX nano-foci making up larger γ-H2AX (super-) clusters [30,33]. Within these 95 nm radii, we observed relatively high degrees of juxta-positioning of 53BP1 and MRE11, each with γ-H2AX (>40%), whereas p-ATM signals were juxtaposed with less than 25% of γ-H2AX signals along a particular damage track (Figure 4D), indicating a dearth of p-ATM signals within the damaged chromatin and possibly hinting at the transitory nature of active ATM at particular chromatin sites or nucleosomes. 

**Figure 6:**
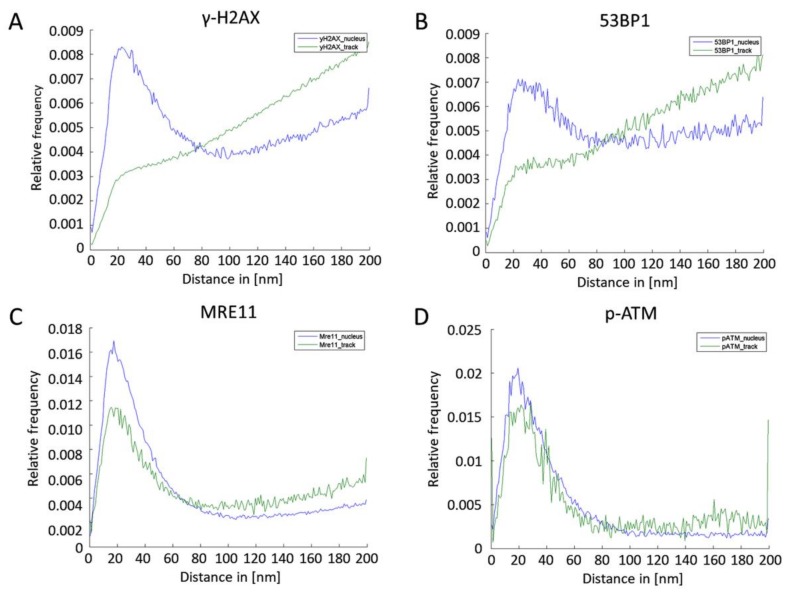
Ripley’s K statistics of γ-H2AX (**A**) and 53BP1 (**B**), MRE11 (**C**) and p-ATM (**D**) signal points locating in the nucleus (blue) or inside the γ-H2AX-defined DNA damage track (green).

By means of Ripley’s distance-frequency analysis, the spatial organization of the labelling tags was studied on the nano- and meso- (µm) scale (Figure 6) inside the track (green curves) and outside the track in the nucleus (blue curves). In case of γ-H2AX, the relative frequency distributions of distances (Figure 6A) showed a peak followed by a linearly increasing curve at greater distances. These shapes are compatible with the formation of clusters of about 80–100 nm (peak at 20–30 nm) embedded in an environment of randomly arranged tags. Outside the track, this clustering was more pronounced, indicating individually dispersed damaged or pan-nuclear off-damage phosphorylated nucleosomes, which could have either been caused by secondary electrons (although this is unlikely, due to their infrequency), or be of native origin, reflecting changing chromatin architecture in alpha-hit cells. Inside the track, there was a large amount of γ-H2AX tags, seems to reflect a dense arrangement of multiple, overlapping individual γ-H2AX chromatin domains along a trajectory. 

The relative distance frequency distribution of 53BP1 (Figure 6B) showed a similar behavior, with the formation of clusters peaking at 20–40 nm. However, there was less clustering outside the damage track, indicating a more uniform distribution of 53BP1 molecules outside the damage-carrying tracks, hinting at an unbound pool of 53BP1 throughout the nucleus. 

In contrast to γ-H2AX and 53BP1, the cluster formation of MRE11 single molecule signals was different (Figure 6C). The cluster sizes were smaller (~60–80 nm with a peak at about 20 nm) and the relative amounts of tags in clusters dominating the distance distributions. This may be related to the MRE11s’ role in processing DSB ends during NHEJ and HR DSB repair [56]. p-ATM signals also appeared to be organized in small clusters (about 60 nm with a peak at about 20 nm) inside and outside the tracks without dispersed signals, especially for the nuclear chromatin outside the track super-foci (Figure 6D).

### 2.4. MRE11 Nano-Cluster Distribution in Alpha Tracks

**Figure 7:**
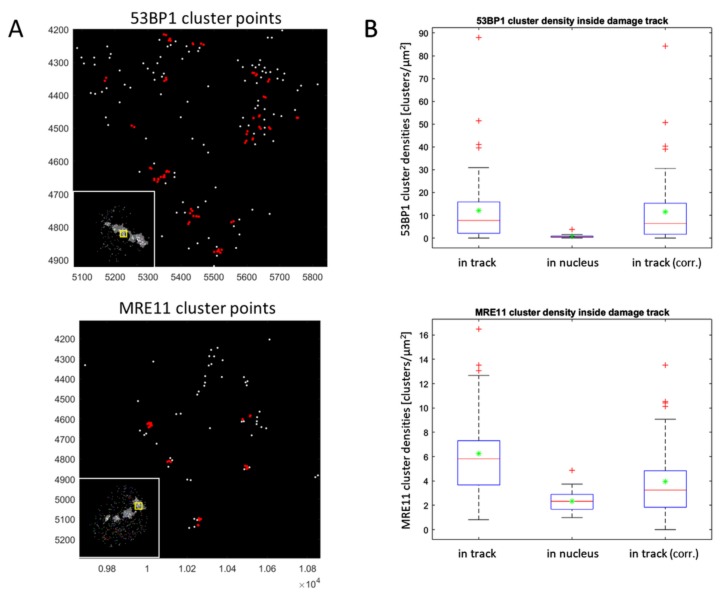
Quantification of cluster densities for 53BP1 and MRE11 inside the average γ-H2AX damage track. (**A**) Distribution of signal point clusters (red) for 53BP1 (top) and MRE11 (bottom) calculated with cluster parameters r_cluster_ = 10 nm and minimum point numbers = 2. The retrieved clusters consisted of predominantly 2–5 signal points in chromatin regions of about 30 nm in diameter. The insets to the lower right display the track and nuclear outline. The X and Y axes give the signal point coordinates. (**B**) Boxplot representation of cluster densities for 53BP1 (top) and MRE11 (bottom) with red lines and green asterisks indicating the median and mean values, respectively. Clusters were quantified inside and outside for each γ-H2AX damage track in a given nucleus. It appears that the density of clusters peaks inside the track, when compared to the remaining nuclear area. Background correction (corr.) of cluster densities (clusters/µm^2^) for the occurrence of pan-nuclear clusters outside the track revealed similar values as specified in Table 1.

Next we determined the number and densities of 53BP1 and MRE11 clusters (Figure 7A) in the nuclear area and inside the alpha-induced γ-H2AX damage tracks (Table 1). The in-track signal densities were corrected for the signal densities encountered in the nuclear chromatin excluding the area of the DNA damage track (Figure 7B). This revealed, as expected, that 53BP1 and MRE11 occur at higher density inside the track compared to the non-irradiated nuclear environment, indicating enrichment at alpha-damaged chromatin (53BP1) and DNA. The MRE11 signal clusters in the nuclear chromatin outside the damage track showed an average density of 2.3 (±0.8; SD) clusters/µm^2^, while the typical γ-H2AX damage track area (3.3 µm^2^ ± 2.0) contained on average 6.3 (±3.4) MRE11 clusters/µm^2^ (Figure 7; Table 1), revealing a 2.7-fold enrichment of MRE11 nanoclusters that may mark repair processes at DSB sites throughout the typical alpha track. 

Using the MRE11 nano-cluster data, we attempted to estimate the potential number of DBSs in an alpha damage track. To obtain such values, we looked at the DSB processive MRE11 protein, which is part of the MRE11-Rad50-Nbs1 (MRN) complex and one of the first DNA repair proteins detected at DSBs in vivo [57] and involved DNA end resection [56]. MRN binds both nucleosomal homoduplex DNA and free DNA ends, and recruits EXO1 for end processing, with 2–4 proteins scanning DNA for or associating with DSB ends [58]. This number aligns with the number of single molecule signals detected in nano-clusters by our analysis. To correct for randomly occurring signals we determined the MRE11 signal densities within the γ-H2AX alpha-trajectories as well as the signal densities in the nuclear chromatin area excluding the γ-H2AX damage track (Figure 7B). Since a DSB will minimally recruit one MRE11 molecule to each DSB end and possibly more to the surrounding DNA, we analyzed our data for MRE11 clusters consisting of 2–5 signal points in regions of about 10–30 nm in diameter. This revealed that, on average, 12 (±9 SD) MRE11 nano-clusters (DSBs) are contained in an average alpha track. Given the limitations of antibody binding and blinking efficiency as well as considering detection losses of signals by camera and software tuning that may lead to >50% signals being missed (M.H. et al., unpublished estimations), this will be a minimal estimate of MRE11-marked DSBs.

## 3. Discussion

High-LET particle irradiation of cells installs dense complex molecular damages of different types along the particle tracks, which may lead to cell death or adverse genomic alterations in the survivors [59,60,61]. DNA repair requires a multi-protein DNA damage response (DDR) that at its center involves HR and/or NHEJ as the main repair pathways [9,56,62]. Here we analyzed the nano-distribution of DDR proteins along alpha particle-induced DNA damage tracks of in-solution Ra-223 alpha-irradiated leukocyte nuclei. The distribution of 53BP1 and MRE11, DDR proteins involved in NHEJ and HR, along the damage track indicates that the cells are proficient for both HRR and c-NHEJ, with the latter being the prominent repair pathway in the resting human G_0_ lymphocytes studied [16,56]. 

The applied internal irradiation scheme [8] has the advantage of generating a clinically more relevant distribution of energy deposition events and avoids the energy loss by mylar foils typically used for alpha irradiation of cells grown on supports (e.g., [52,63]) and generates cells with single alpha hits in which the γ-H2AX-positive alpha tracks lay parallel to the optical plane, facilitating SMLM analysis. Although SMLM has been established as a powerful technique for measuring molecular architectures in cellular systems on the nano scale, further aspects need to be considered in relation to the data interpretation. The resolution of about 15 nm determines the smallest distance between two blinking fluorescent points. The fluorescence molecules, however, are part of an antibody system of primary and secondary antibodies with typical sizes of 20–25 nm. Thus, fluctuations of these antibodies would have an influence on the results of distance measurements. To overcome this statistical shortcoming, fluctuations or drifts of signals have to be avoided during the measurement of a cell nucleus. By advanced thermal stabilization in the mK range of the microscopic setup resulting in a drift of only a few nm (for details, see [36,39]) and careful fixation of the specimen (see Material and Methods) this principle requirement was fulfilled. Nevertheless, the labelling scheme and the dimensions of the labelling molecules have to be considered if parameters for presumptive co-localization of two target sides have to be determined. This is the reason for the choice of the 95-nm co-localization distance as an upper estimate between two fluorescence molecules attached to closely positioned target molecules. 

Based on such in principle considerations of experimental design and realization, we followed the distribution of the activated DSB-responsive ATM kinase that is activated after NBS1 (ATM) binding to a DSB [17,18]. Active, monomeric ATM phosphorylates, among other downstream targets, the histone 2 variant H2AX at its Serine 139 moiety, forming γ-H2AX [21]. The latter is in various cell types the most prominent and persisting chromatin mark along alpha particle and accelerated ion tracks [31,51,53,64,65]. 

The SMLM analysis of DNA damage-responsive proteins along the γ-H2AX-marked damaged chromatin tracks revealed that the damage track often consisted of super-foci of variable size along the tracks that contained numerous γ-H2AX histone single-molecule signals in variable density indicating a heterogeneous nano-architecture of γ-H2AX-positive chromatin, as has also been observed for other high-LET particle irradiation [23,30,33]. The distribution of γ-H2AX along proton tracks has been reported to involve foci sizes for γ-H2AX and 53BP1 of about 540 nm, with the nanostructure subunits in the tracks being about 130 nm [31], while Lopez-Perez et al. observed 100 nm subunits [30], both after C ion irradiation at the Munich tandem accelerator SNAKE (energy in the cell layer: ~27 MeV, LET ~500 keV µm^−1^). Hence, the differences between the two studies are likely due to differences in the optical systems and the resolution limits they used. Using an external alpha irradiation device with an Am-241 source, Reindl et al. observed large 53BP1 clusters (super-foci) along damage tracks in the µm range [66]. The 53BP1 nano-foci located within the γ-H2AX environment showed only partial co-localization, compatible with our findings for heavy N ions [28] and the results after C ion irradiation [31]. The super-foci (cluster) sizes observed for γ-H2AX and 53BP1 in our alpha particle investigations are in agreement with the view that both proteins are part of the broader chromatin environment of DSBs, likely facilitating DSB repair [24,27,34]. We also found that 53BP1 and MRE11 signals were located in the non-irradiated nuclear environment, while their frequency was enriched in the alpha-damaged chromatin tracks, suggesting that the damage caused by a single alpha particle did not deplete the protein pool of a hit nucleus, whereas 53BP1 depletion was observed after repeated C ion irradiation [67]. The fact that the alpha damage tracks contained 60–70% of all γ-H2AX point signals of a nucleus, while less than 30% of 53BP1, MRE11 or p-ATM signals were located inside γ-H2AX damage tracks, may furthermore be related to tech variables like antibody performance, with the 53BP1 and MRE11 antibodies used were detecting the entire protein pool in a nucleus off and on the damaged chromatin areas, while the phospho-specific anti γ-H2AX and p-ATM Abs likely detecting only the active proteins. The dispersed p-ATM signals therefore likely represent active kinases throughout the nuclei that may induce the pan-γ-H2AX signal in alpha-hit cells noted previously [53,54]. 

In contrast to the broad distribution of 53BP1 and γ-H2AX along the alpha-tracks MRE11 and p-ATM showed a smaller range of nano-clustering. Within 95 nm radii, we observed juxtapositioning of p-ATM single molecule signals with a quarter of the γ-H2AX signals detected in an average alpha-damaged chromatin track, which may hint at a transitory action of active ATM at particular nucleosomes in damaged chromatin. Furthermore, p-ATM signals appeared to form small clusters of about 60 nm, suggesting at the action of a few closely spaced p-ATM molecules acting on closely spaced nucleosomes. Furthermore, there remains the possibility that the nano-clusters reflect active ATM molecules bound to DNA flanking DSBs [68]. 

MRE11 on the other hand, is part of the MRE11-Rad50-Nbs1 (MRN) complex and one of the first DNA repair proteins detected at DSBs in vivo [57] and forms micro-domains (single foci) in γ-H2AX chromatin domains after high LET irradiation [69]. MRN binds both nucleosomal homoduplex DNA and free DNA ends, activates ATM [49,70] and recruits EXO1 for end processing [58]. Interestingly, MRN (MRE11) seems to play an important role in both the NHEJ and HR DSB repair pathways (reviewed by [56]). We chose MRE11 for SMLM analysis because of antibody performance and its being involved in both HR and NHEJ, with c-NHEJ being the major DSB repair pathway in G1 or G_0_ cells like the human leukocytes analyzed in this contribution, while a-NHEJ seems to play a minor role in normal human cells [19,20]. MRE11 is known for being associated with DSB ends in the range of 2–4 proteins [58]. SMLM revealed that MRE11 forms nano-clusters of about 2–5 single molecule signals embedded in the γ-H2AX chromatin. Assuming that those nanoclusters mark DSB ends that contain MRN (MRE11) molecules engaged in DSB processing and correcting for the background outside the damage tracks, we observed a 2.7-fold enrichment of MRE11 nanoclusters that likely mark repair processes at DSB sites. At a density of on average 6.3 ± 3.4 (SD) MRE11 clusters/µm^2^, this suggests the presence of 11.6 ± 8.8 MRE11 nanoclusters (DSBs) per track. However, our estimates on DSB numbers via MRE11 nanoclusters and area calculations are surely giving minimal numbers, as signals may be lost during the bleaching steps [71,72], and not all DSBs may have recruited or involved MRE11 for processing. Nonetheless, the derived DSB numbers are in the range of those determined for externally irradiated alpha particle-induced γ-H2AX foci by wide-field microscopy [11,52] or those obtained with high-resolution confocal microscopy [1]. 

Estimations on DSB numbers may hypothetically be derived based on observations that γ-H2AX super-focus formation involves approximately a megabase chromatin domain around a single DSB [22,23], and the fact that this domain displays a radius of about 250 nm. The γ-H2AX damage track lengths we measured in leukocyte nuclei were about 3–5 µm with widths of 1–1.5 µm. Calculating the area of an alpha-trajectory in our SMLM point matrices and using the approximation of 1 DSB/250 nm radius of γ-H2AX-marked damaged chromatin in a diploid G1 nucleus, this assumption also revealed that about 12 DSBs may be contained in the damaged area of an average γ-H2AX alpha particle track of 1.27 µm^2^ in a lymphocyte nucleus of 35 µm^2^; a frequency that aligns with our MRE11 data. It is of note that the observed and calculated DSB numbers are likely minimal ones, since high LET-induced stacked DNA damages and breaks may lead to smaller distances between DSBs. 

Alternatively, one may hypothetically calculate the DSB yield under the assumption of 1 DSB focus/1 MB of DNA [22,23,32]; doing so, it is suggested that about 300 DSBs may be contained in an average alpha track of a leukocyte nucleus. Leaving alone the uneven distribution of DNA in different nuclear micro-environments, this number seems to align with the EM study of Lorat et al., who detected micro-clusters of pKu70 DNA repair proteins along carbon ion-induced (9.5 MeV/n, LET = 190 keV µm^−1^) damage tracks that suggest that up to 500 DSBs/µm^3^ may be contained in such a track [34]. In the former study Ku70 was observed to form nano-clusters of 2 or 4 p-Ku70 gold signals along the chromatin tracks damaged by particle irradiation. Since we failed to obtain successful pKu70 immuno-signals suitable for SMLM analysis, it will be interesting to further study NHEJ components in alpha-damaged chromatin tracks. Other approaches also suggest the presence of numerous and clustered DSBs [73] and short DNA fragments along high-LET tracks [74]. Du et al. calculated 140 DSB along a 10 µm C ion track [64]. Alpha particles; however, have a shorter range and deposit much of their energy near the track end [13,75]. Still, it has to be considered that in the case of heavy ions, the absorbed dose was at least one order of magnitude larger than in our experiments, which may speak for the estimate based on our MRE11 SMLM measurements. Nevertheless, these results indicate that further investigations may be necessary not only to learn more about the number of DSBs induced, but also to better understand the impact of chromatin nano-architecture and repair-related nano-scale protein arrangements for the fidelity of DNA damage repair and its pathways. 

## 4. Materials and Methods

### 4.1. Blood Sampling, Irradiation and Cell Isolation

The white blood cells from peripheral blood samples were from healthy volunteers obtained during a previous study by Schumann et al. [8]. The research was approved by the ethics committee of the Medical Faculty of the University of Würzburg, Germany (Az: 165/14). 

White blood cells were isolated by density centrifugation, irradiated in-solution and stored in 70% Ethanol at −20 °C until immunostaining. Briefly, blood was collected using Li-Heparin tubes (S-Monovette^®^, Sarstedt, Germany) and homogeneously irradiated in solution with Ra-223 dichloride solution (Xofigo^®^, Bayer, Germany) diluted in phosphate buffered saline (PBS) to result in 100 mGy absorbed dose after 1 h of irradiation as described by Schumann et al. [8]. After internal irradiation, leukocytes were isolated from the Ra-223-containing blood solution using density centrifugation in CPT Vacutainer tubes (BD, Germany) according to the manufacturer’s instructions. Finally, isolated cells were washed twice in PBS and fixed in ethanol at a final concentration of 70%, followed by storage at −20 °C until immunostaining.

### 4.2. Calculation of the Absorbed Dose to the Blood

The calculation of the absorbed dose to the blood was achieved by quantification of the activity in the blood sample using a calibrated, high-purity germanium detector and an absorbed dose coefficient of 15.5 mGy∙kBq^−1^ for 1 mL of blood at 1 h irradiation; for details see Schumann et al. [8].

For this study, a cell sample with a nominal absorbed dose of 100 mGy was chosen, since such a sample displays numerous cells with single alpha hits and the preferred damage tracks parallel to the optical plane [8].

### 4.3. Immunofluorescent Staining

Immunofluorescent DSB and repair protein staining has been described in detail elsewhere [36,76]. In brief: the cells fixed in ethanol were subjected to cyto-centrifugation followed by immunofluorescent staining to detect DNA damage-associated protein accumulation as microscopic foci at DNA double-strand break sites as previously described [77]. Primary antibodies against γ-H2AX (Mouse anti-γ-H2AX; Merck Chemicals), rabbit anti-MRE11 (Novus), rabbit anti-phospho-ATM (pS1981; abcam), and 53BP1 (Rabbit anti-53BP1; Novus) were applied and detected with secondary goat anti-mouse Alexa-488 (Mobitec, Göttingen, Germany), goat-anti-rabbit-Cy5 or goat-anti-mouse Cy3-labeled antibodies (both Dianova). For SMLM, preparations were embedded in ProLong Gold anti-fade solution (Thermo Fisher Scientific, Schwerte, Germany). Wide field fluorescence images were recorded using a Zeiss Axioimager 2i fluorescence microscope system (ISIS; MetaSystems, Altlussheim, Germany). Cells that showed deformed or overlapping nuclei were excluded from analysis. 

### 4.4. Single-Molecule Localization Microscopy (SMLM)

The microscopic setup has been described in detail elsewhere [36,41,44]. The motorized inverted TILL Photonics FEI microscope is equipped with four lasers (excitation wavelength/maximum power: 405 nm/120 mW; 491 nm/200 mW; 561 nm/200 mW; 642 nm/140 mW (not used in this study)), altogether assembled in a LightHub laser combiner (Omicron Laserprodukte GmbH, Rodgau-Dudenhofen, Germany). Excitation laser wavelength and intensity are set by a polychromatic AOTF (AA Opto Electronic, Orsay Cedex, France). A variable beam expander 10BE03-2-8 (Standa ltd, Vilnius, Lithuania) and a Flat-Top-Profile forming optics PiShaper (AdlOptica GmbH, Berlin, Germany) are used to expand and homogenize the laser beam. An achromatic focusing lens (f = 250 mm) and a 100× /NA 1.46 oil plan apochromatic objective lens (Carl Zeiss Microscopy, Göttingen, Germany) are used to project the circular Flat-Top laser beam profile into the object plane. Emission and excitation paths are separated by two quadband interference filter glasses F73-410 and F72-866 (AHF analysentechnik AG, Tübingen, Germany). Emission light is further projected (magnified) by the objective and tube lenses (Carl Zeiss Microscopy, Göttingen, Germany) and an additional twofold expander. Signals were detected with an iXon Ultra Andor electron multiplying charge-coupled device (EMCCD) camera (Andor Technology, Belfast, Northern Ireland). 

For SMLM image acquisition, the lasers were set to maximal powers (491 nm at 2.52 kW/cm^2^; 561 nm at 4.65 kW/cm^2^) and 2000 frames were acquired with an EM-gain of 100 and 100 ms exposure time per frame with each excitation laser wavelength. 

### 4.5. SMLM Data Analysis

In-house software (for detailed descriptions see [40,44,78,79,80,81]) written in MATLAB was used to calculate signal point localizations from blinking events in raw image stacks. In brief, background signals were excluded, and the two-dimensional intensity profile of each fluorescent signal was subjected to Gaussian fitting through the whole image stack. The resulting peak positions correspond to the localization coordinates. These coordinates are stored in a data matrix along with localization errors estimated for each individual point and other additional information. The data matrix was used to compute super-resolution localization images that visualize each signal coordinate at a pre-defined resolution (pixel size = 10 × 10 nm^2^) and with a Gaussian blur according to the individual localization error. 

In-house software (for detailed descriptions see [40,44,78,79,80,81]) written in MATLAB was used to calculate signal point localizations from blinking events in raw image stacks. In brief, background signals were excluded, and the two-dimensional intensity profile of each fluorescent signal was subjected to Gaussian fitting through the whole image stack. The resulting peak positions correspond to the localization coordinates. These coordinates are stored in a data matrix along with localization errors estimated for each individual point and other additional information. The data matrix was used to compute super-resolution localization images that visualize each signal coordinate at a pre-defined resolution (pixel size = 10 × 10 nm^2^) and with a Gaussian blur according to the individual localization error. 

Cells were manually selected and only those with clearly visible γ-H2AX stained damage tracks were used for further analysis. Furthermore, overlapping damage tracks and tracks in too close proximity to each other were also sorted out. 

### 4.6. Masking of SMLM Data

The coordinate matrices of γ-H2AX and of the co-stained partner protein (53BP1, MRE11, or p-ATM) were further masked to reduce localization signals to those detections (=foci) clearly belonging to γ-H2AX damage tracks. In all, two different masking methods were applied either manually or semi-automatically, based on density. 

In the first approach, resulting localization images of γ-H2AX were manually processed into masks that discriminate between background and damage track. In all cases, a slightly larger region around the γ-H2AX damage track was used as mask to prevent exclusion of potentially relevant signals of partner proteins. 

In the second approach, we performed DBSCAN [82] analysis on the γ-H2AX coordinate matrix to determine the damage track and foci masks. DBSCAN (Density Based Spatial Clustering of Applications with Noise) was designed to determine clusters in a spatial database. For each point of a cluster the neighborhood within a given radius has to contain at least a minimum number of other points. Ideally, the appropriate parameters (radius and minimum number of points) of each cluster and at least one starting-point from a given cluster have to be known. Then, all points that are density-reachable from the given starting point can be retrieved. Since this information is usually not available in advance for all clusters of the database, a simple and effective heuristic can be applied to determine the parameters of least dense cluster in the database. Therefore, DBSCAN can use global, i.e. the same values for all clusters. These global parameter values are specifying the lowest density which is not considered to be noise. Global parameters used for this analysis are a minimum number of points depending on the specimen (=12 for γ-H2AX + 53BP1; 25 for γ-H2AX + MRE11/p-ATM) inside the radius ε = 200 nm. If an individual point fulfilled these parameters, it was included in a focus. From the point pattern determined by this procedure, an image was generated showing the convex hull of the point patterns that was further used as mask for γ-H2AX and partner protein localization signals included into a focus. The resulting masked coordinate matrices were used for all subsequent analyses. 

### 4.7. Tracklength Estimation

For each track, a start and end point was defined by manually masking signal coordinates. On an axis through a γ-H2AX damage trajectory defined by this operation, a reference point was defined at the outermost position. From that reference point, the minimum and maximum Euclidean distances among all γ-H2AX signal point coordinates were calculated. These two values served as the start and end point measurements of the track, and the difference between those two distances was considered to be the length of the track.

### 4.8. Cluster Analysis 

We performed DBSCAN [82] cluster analysis on the coordinate matrices of γ-H2AX and repair proteins. Optimized parameters used for cluster analysis were a minimum number of points = 2 inside the radius r_cluster_ = 10 nm. These parameters were derived from pronounced distance clustering detected by the nearest-neighbor analysis of γ-H2AX and repair protein SMLM data. 

### 4.9. Ripley’s Distance Frequency Analysis

Ripley’s-based approaches (for details see [40]) estimate the point-to-point distances by direct measurement of distances from the central point to the peripheral ones. The graphical display of the Ripley’s point-to-point distance information without regarding absolute position information makes it possible to discriminate between specimen structure signals and background signals. The original curve shows noise-like fluctuations due to the superposition of the specimen signals, the blurring of the microscope transfer function and the camera chip acquisition characteristic. In total, these parameters restrict the possible localization precision of a point-like fluorescence burst signal. The envelope function of the distance frequency histogram can be divided into one or several functions which represent particular distance accumulations. In this way, elements of an organized specimen structure can be identified and subjected to further computation and classification. In addition, the appearance of spatially disorganized background signals tends to approximate a monotonically increasing linear function. The specimen structure related to non-random local clustering appears as individual peaks of the histogram envelope function. The maximum value of such a peak indicates the characteristic distance of a specimen structure. The symmetry and width of the peak corresponds with molecular conformation and spatial organization of the labelled elements.

### 4.10. Co-Localization Analysis

The “co-localization” between repair protein 53BP1, MRE11 or p-ATM repair protein signal points with γ-H2AX signal points was calculated by estimating the spatial volume of the protein-antibody construct used for tagging. Assuming the space necessary for stoichiometric reasons, “co-localization” can only be defined by a co-localization distance, which was estimated at 95 nm on average. This distance value needs to be fulfilled by the distance between a repair protein signal and a γ-H2AX signal point. First, the distance between each repair protein signal point to all γ-H2AX points was calculated and the relative amount of protein γ-H2AX pairs with distances within the co-localization distance was determined. Every γ-H2AX signal is only allowed to co-localize with one repair protein signal, but one repair protein signal can co-localize with multiple γ-H2AX signal points.

## 5. Conclusions

In all, our study supports the presence of numerous DSBs in the nano-environment of γ-H2AX-positive chromatin of the high LET alpha-induced DNA damage tracks in leukocyte nuclei. When performing next neighbor analyses of heterologous distances for γ-H2AX with 53BP1, MRE11 and p-ATM and cluster analyses of homologous distance frequencies, we found typical distance ranges of 60–80 nm relevant for the repair processes, which are in good agreement with the ~50 nm–100 nm clusters and close juxtapositioning of γ-H2AX and 53BP1 molecules detected by EM ultra-resolution techniques [34,65] that may aid and direct the local DNA damage response. Although our algorithms for point distribution analyses based on geometry and distance parameters suggest a relatively heterogeneous arrangement of phosphorylation sites and repair proteins, the cluster analyses give hints for a given nearly constant order of magnitude in size for the distribution of proteins. As shown earlier by GFP-tagged H2B and radial density function calculations [83], the nucleosome patterns obtained from Hela cells or fibroblasts were not randomly organized within units of about 300 to 400 nm size. Based on this assumption, one might speculate that the nano-arrangements of γ-H2AX and repair proteins may follow similar rules of genome architecture organization. Considering homeomorphisms and scale invariances within the detected γ-H2AX and repair protein clusters should therefore reflect topological patterns with high degrees of similarities. Recent results using persistent homology as an approach for SMLM data analysis and classification of γ-H2AX clusters after photon irradiation have revealed a dose-independent topological similarity of clusters depending on the neighborhood to heterochromatin [45]. The application of such approaches in future investigations will give further insights into complex damage processes and the relevance of chromatin architecture for repair process propagation. 

## Figures and Tables

**Table 1:** Quantification of 53BP1 and MRE11 nano-cluster densities per average γ-H2AX damage track.

Variable	53BP1				Mre11		
	Mean	SD	SE		Mean	SD	SE
N_cluster/track_	17.5	33.3	4.5	-	19.3	12.0	1.9
N_clusters/nucleus_	23.8	19.2	2.6	-	140.3	54.9	8.7
N_clusters/track_ (corrected)	**16.5**	32.0	4.4	-	**11.6**	8.8	1.4
A_track_	1.3	1.0	0.1	-	3.3	2.0	0.3
A_nucleus_	34.5	6.5	0.9	-	63.9	12.5	2.0
ρ_cluster/track_	12.2	15.5	2.1	-	6.3	3.4	0.5
ρ_cluster/nucleus_	0.7	0.6	0.1	-	2.3	0.8	0.1
ρ_cluster/track_ (corrected)	**11.5**	15.1	2.0	-	**3.9**	3.1	0.5

N: Average number of clusters; A: Area in [µm^2^]; ρ: Density of clusters [1/µm^2^]; SD: Standard deviation; SE: Standard error of the mean; r_cluster_ = 10 nm, minimum point numbers = 2. Bold numbers represent values corrected for the off-track background of a nucleus.
